# Aetiology, susceptibility and outcomes of fever in patients receiving chemotherapy in Malawi: a prospective study

**DOI:** 10.1093/jacamr/dlae173

**Published:** 2024-10-29

**Authors:** Kaushik Puranam, Meagan Harrington, Edwards Kasonkanji, Gerald Tegha, Maria J Chikasema, Mena L Chawinga, Maganizo B Chagomerana, Robert Krysiak, Satish Gopal, David van Duin, Melissa B Miller, Matthew S Painschab

**Affiliations:** Department of Medicine, Duke University, Durham, NC, USA; University of North Carolina Project-Malawi, Cancer Program, Lilongwe, Malawi; University of North Carolina Project-Malawi, Cancer Program, Lilongwe, Malawi; University of North Carolina Project-Malawi, Cancer Program, Lilongwe, Malawi; University of North Carolina Project-Malawi, Cancer Program, Lilongwe, Malawi; University of North Carolina Project-Malawi, Cancer Program, Lilongwe, Malawi; University of North Carolina Project-Malawi, Cancer Program, Lilongwe, Malawi; Department of Medicine, University of North Carolina at Chapel Hill, Chapel Hill, NC, USA; University of North Carolina Project-Malawi, Cancer Program, Lilongwe, Malawi; Department of Medicine, University of North Carolina at Chapel Hill, Chapel Hill, NC, USA; Center for Global Health, National Cancer Institute, Rockville, MD, USA; Department of Medicine, University of North Carolina at Chapel Hill, Chapel Hill, NC, USA; Department of Pathology and Laboratory Medicine, University of North Carolina at Chapel Hill, Chapel Hill, NC, USA; University of North Carolina Project-Malawi, Cancer Program, Lilongwe, Malawi; Department of Medicine, University of North Carolina at Chapel Hill, Chapel Hill, NC, USA

## Abstract

**Objectives:**

To evaluate causes of fever, including resistance patterns, in patients undergoing cancer treatment in Malawi.

**Methods:**

In this prospective cohort study, enrolled patients undergoing chemotherapy at Kamuzu Central Hospital in Lilongwe, Malawi were given a thermometer. If a temperature of ≥38°C was recorded, they were instructed to return for hospitalization, standardized fever workup, and antibiotics. All patients were followed through 90 days post-fever event or completion of chemotherapy.

**Results:**

One hundred and five patients were screened and 50 were enrolled. Of the enrollees, 26 (52%) were men and 26 (52%) were HIV positive, with a mean ART duration of 7 years and CD4 count of 293 cells/µL. The most common diagnoses were aggressive non-Hodgkin lymphoma (40%) and Hodgkin lymphoma (22%). Twenty-three febrile events were recorded from 15 patients. Of the 23 events, a causative agent was isolated in 13 cases: *Escherichia coli* (6), *Plasmodium falciparum* (3), *Streptococcus pneumoniae* (2), *Pseudomonas aeruginosa* (1) and *Citrobacter freundii* (1). Of the six *E. coli* isolates, all were found to be resistant to fluoroquinolones and 4/6 (66%) were resistant to cephalosporins. All patients but one survived; the death was attributed to *Pseudomonas* bacteraemia.

**Conclusions:**

This study describes laboratory-confirmed causes of neutropenic fever (NF) in cancer patients in Malawi. Gram-negative rods, followed by malaria, were the most common source of infection. Gram-negative rods were associated with high rates of antimicrobial resistance. Malaria and resistant bacterial infections should be considered for NF treatment and prevention in sub-Saharan Africa.

## Introduction

Cancer incidence and treatment is expected to rise substantially in sub-Saharan Africa (SSA). In 2022, there were an estimated 847 974 new cases of cancer in SSA, with 558 878 deaths.^1^ Due to relatively high rates of HIV, population growth and ageing, incidence of cancer in SSA is expected to reach 1.5 million, with over 1 million deaths annually, by the year 2040.^2–5^

With the cancer burden in SSA escalating, the occurrence of treatment-related infections is of increasing concern. Patients with cancer are particularly vulnerable to infections due to multiple factors, including cancer-related neutropenia or immunoglobulin deficiencies and frequent interactions with healthcare settings.^6^ Moreover, the coinfection rate of HIV among cancer patients in SSA can be as high as 35%, further exacerbating the risk of infections.^7^ Chemotherapy-associated neutropenic fever (NF) represents a critical complication in cancer patients, with over 80% of haematological malignancy cases and 10%–50% of solid tumour cancer patients experiencing NF.^8,9^ Alarmingly, mortality rates associated with NF can reach 10%.^10^ Timely identification and intervention are essential in managing NF, as it represents an oncological emergency that necessitates immediate action to prevent fatal outcomes.

In high-income countries (HICs), NF guidelines aimed at prompt empirical anti-infective prophylaxis have been validated and shown to improve patient outcomes.^11,12^ However, in SSA, little is known about neutropenia-related deaths during cancer treatment. Most deaths occur at home or at facilities without capacity for blood cultures, leaving no ability to identify an infectious aetiology. In SSA, pathogens in cancer populations are likely different from the HIC context due to frequent co-occurrence of HIV, absence of central venous catheters (CVCs), different antibiotic use and availability patterns, including frequent over-the-counter use without physician prescription, different endemic infections and exposures (e.g. malaria, TB) and different cancer treatment intensity and regimens. Furthermore, the regional bacterial spectrum and resistant patterns of SSA neutropenic patients likely differ as well,^13,14^ with multiple recent reports of highly drug-resistant bacterial infections across the region.^15,16^ Given these differences, it is not possible, nor sensible, to extrapolate HIC NF guidelines for use in SSA.

Few published studies in SSA have investigated the frequency of NF and aetiology of bacteraemia in patients undergoing high-risk chemotherapy, with few studies including outcome data for patients.^16–20^ A better understanding of NF and the common bacterial infections among cancer patients in SSA is needed for safer cytotoxic therapy escalation, ultimately leading to improved cancer-specific mortality and morbidity. By defining the spectrum of bacterial infections and associated antimicrobial resistance (AMR) during NF events in cancer treatment, better prophylaxis and treatment plans for cancer populations across SSA can be developed. Herein we describe a prospective cohort of cancer patients undergoing chemotherapy who received detailed evaluation of NF episodes in Lilongwe, Malawi.

## Methods

### Study design

We performed a prospective, longitudinal study between March 2019 and July 2020. Cancer patients undergoing chemotherapy at Kamuzu Central Hospital (KCH) were followed to identify protocol-defined fever episodes, which received a standardized evaluation. KCH is the national teaching hospital and serves as the main cancer referral centre for over 10 million people in Malawi.

### Participant procedures

Participants were included who were initiating chemotherapy and had either (i) HIV and ANY biopsy-proven cancer; or (ii) ANY haematological malignancy, regardless of HIV status. In addition, participants needed to be ≥18 years old and live within a 100–150 km radius of KCH. Enrolled patients were given a thermometer and fever log to measure and record their temperature twice daily, with instructions to return to the clinic if a temperature was recorded to be ≥38.0°C. Fever evaluation included a complete blood count with differential, two blood cultures from different arms, malaria rapid diagnostic (antigen) test (MRDT), chest radiograph, urinalysis with urine culture, and additional tests as dictated by symptoms and clinician discretion. All patients were admitted to the hospital and treated with the local standard-of-care antibiotics including empirical ceftriaxone 2 g IV daily and ciprofloxacin 750 mg twice daily or infection-specific antibiotic regimens when a specific infection was suspected or confirmed. Blood cultures, if positive, were obtained approximately every 48–72 h until sterile, and antibiotics were tailored based on culture and antimicrobial susceptibility results.

Febrile patients were followed for 90 days post-fever for outcomes, largely in the outpatient setting. Patients were included more than once if they had another fever event after remaining afebrile for ≥72 h. Those who remained afebrile were followed through to the completion of chemotherapy. The definition of neutropenia varies across the literature and the risk of infection increases significantly as the absolute neutrophil count (ANC) decreases below 0.5 × 10^3^ cells/µL (severe neutropenia). For purposes of this study, neutropenia was defined as having an ANC below 1.5 × 10^3^/µL, in part due to the delay in laboratory value turnaround time in our setting, as well as limits on frequency of laboratory testing that we were able to complete, which likely limits our sensitivity in detecting trough ANC values. For the purposes of this study, all fever-related admissions were included for evaluation, regardless of ANC value.

### Laboratory procedures

All microbiological testing for this study was completed at the University of North Carolina Project Malawi laboratory, certified by College of American Pathologist Routine Microbiology Combined External Quality Assurance. The laboratory has been certified for numerous multinational clinical trial groups including the NIH HIV Prevention Trial Network. Positive aerobic blood cultures were subcultured on sheep blood agar and MacConkey agar plates. Isolates were identified manually using standard analytic profile index testing or by automated organism identification utilizing the BD Crystal ID system Enteric kit. Antimicrobial susceptibility testing was performed on Mueller–Hinton media incubated at 35°C and interpretation of the antibiotic disc zone size measurements was done in accordance with CLSI guidelines. Multidrug resistance (MDR) was defined as resistance to at least one antibiotic in three different antimicrobial classes, as per the US CDC definition. We did not have the capacity to do PCR testing for mechanisms of resistance in real time.

### Statistical methods

Baseline statistics were summarized using mean and standard deviation if normally distributed, and median and IQR if non-normally distributed, and proportions for categorical variables. We used proportions to summarize the frequency of isolated infectious pathogens. Differences in the spectrum of bacteria and clinical outcomes between subpopulations were assessed using Fisher’s exact test, given the small number of events. Mortality among patients who developed fever was assessed from the day of fever through to 90 day follow-up.

### Study data

Study data were collected and managed using REDCap electronic data capture tools hosted at the University of North Carolina at Chapel Hill.^21,22^

## Ethics

This study received ethical approval from UNC IRB (18-2285) and NHSRC (2173).

## Results

### Baseline patient characteristics

**Figure 1.**
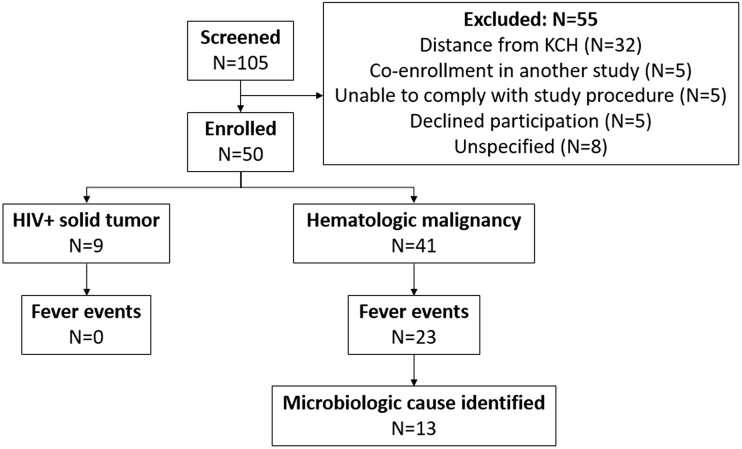
Flowchart of patient screening, enrolment and exclusion criteria. In total, 105 patients were screened and 50 were enrolled. We excluded 55 patients who were unable or unwilling to comply with study procedures, were co-enrolled in another study, or lived >150 km from KCH due to availability of closer facilities for treatment and transport challenges. Of the 50 enrolled patients, 9 were HIV+ with a solid tumour, and 41 had a diagnosed haematological malignancy. Twenty-three fever events were noted in the haematological malignancy group, from which 13 cultures were isolated for further evaluation.

We screened 105 patients and enrolled 50. Out of the 55 patients not enrolled in the study, the primary reason for screening failure was distance from KCH (32; 57%), followed by co-enrolment in another study (5; 9%) (Figure 1).

Median participant age was 44 years (range: 19–75 years) and 26 (52%) were men (Table 1). Twenty-six (52%) participants were HIV positive with a mean ART duration of 7 years and CD4 count of 293 cells/µL. The most common haematological malignancies were diffuse large B cell lymphoma (DLBCL) and Hodgkin lymphoma, and the most common solid tumours were breast cancer and cervical cancer. The full list of diagnoses is shown in Table 1. Among haematological malignancies, 11 (27%) were Ann Arbor Stage III and 10 (24%) were Ann Arbor Stage IV.

**Table 1.** Baseline characteristics of 50 patients initiating chemotherapy in Lilongwe, Malawi

Characteristic	
Baseline clinical characteristics	
Male, *n* (%)	26 (52)
Age (years), mean (SD)	44 (13)
HIV, *n* (%)	26 (52)
Weight (kg), median (IQR)	56 (51–62)
BMI (kg/m^2^), median (IQR)	21.6 (19.3–23.4)
Time since HIV diagnosis (years)	7.8
Time on ART (years)	6.8
Diabetes, *n* (%)	1 (2)
Alcohol use, *n* (%)	6 (12)
Smoking use, *n* (%)	5 (10)
History of KS, *n* (%)	2 (4)
Previous chemotherapy or radiation, *n* (%)	4 (8)
Diagnosis	
DLBCL, *n* (%)	11 (22)
Hodgkin lymphoma, *n* (%)	11 (22)
Low-grade B cell lymphoma, *n* (%)	5 (10)
Burkitt lymphoma, *n* (%)	4 (8)
Multicentric Castleman disease, *n* (%)	3 (6)
Breast, *n* (%)	3 (6)
T cell non-Hodgkin lymphoma, *n* (%)	3 (6)
Cervical, *n* (%)	2 (4)
Non-Hodgkin lymphoma, NOS, *n* (%)	2 (4)
Other, *n* (%)	6 (12)
Baseline laboratory results	
Bilirubin (mg/dL), mean (SD), [*n*]	1.4 (3.8) [44]
CD4 count (cells/µL), mean (SD), [*n*/*N*]	293 (240) [23/26]
Creatine (mg/dL), mean (SD)	0.9 (0.5)
Haemoglobin (g/dL), mean (SD)	10.1 (2.7)
Platelet count (×10^9^/L), mean (SD)	350 (222)
WBC count (×10^9^/L), mean (SD)	18.7 (30.8)
Neutrophil count (×10^9^/L), mean (SD)	6.1 (7.1)

KS, Kaposi’s sarcoma; NOS, not otherwise specified.

Infectious prophylaxis was common. Granulocyte colony-stimulating factor (G-CSF) was administered to 26% of all patients and 30% of HIV-positive patients during their treatment course; ciprofloxacin prophylaxis was used in 99/304 (32%) chemotherapy cycles, and no CVCs were used. Despite routine recommendation of trimethoprim/sulphamethoxazole prophylaxis for people living with HIV, this was not frequently employed in this population, with only 24/304 chemotherapy cycles including trimethoprim/sulphamethoxazole as a concomitant medication.

### Febrile events

We observed 23 independent febrile episodes from 15 patients. Two patients had four febrile events each, one patient had three febrile events and the rest had one event each. Twelve febrile events were associated with neutropenia. The other 11 events were associated with a reduced neutrophil count compared with normal, though it did not meet the definition of neutropenia.

When admitted, patients displayed a variety of symptoms. The most common symptom was headache, seen in 14 admissions. An additional six cases of abdominal pain were reported, alongside six patients with a cough, and two with shortness of breath. Of note, for chest imaging, there were 21 chest radiographs and two CT scans done. Abnormalities detected included two patients with pleural effusions and one lobar consolidation. Vital signs for fever admissions [median (range) for each] were as follows: temperature 38.7°C (38°C –39.7°C); heart rate 95 beats per minute (77–126); systolic blood pressure (SBP) 110 mmHg (81–155); diastolic blood pressure 69 mmHg (46–86); respiratory rate 22 breaths per minute (16–30); and oxygen saturation 98% (88%–100%). Of these febrile events, 5/23 (22%) presented with SBP of ≤100 mmHg, 14/23 (61%) with respiratory rate of ≥22 breaths per minute, and 2/23 (9%) with encephalopathy. Only 4/23 (17%) presented with two of the three quick SOFA (qSOFA) parameters being positive, which has been associated with increased risk for in-hospital mortality. Lactate measurements and blood gases were not available. Patients presented to hospital a median of 7 h after fever initiation, with antibiotics starting a median of 1 h after arrival at hospital.

### Isolates and susceptibility

Of the 23 blood and urine cultures obtained from febrile episodes, 13 were positive for a pathogen (Table 2). Of these, 10 (77%) were found to be bacterial and three (23%) were malarial, two of which were detected by MRDT and one by peripheral blood film after a negative MRDT. Eight isolates were Gram-negative bacteria and two were Gram-positive *Streptococcus pneumoniae*. Of the Gram-negative bacteria, six were *Escherichia coli*; *Pseudomonas aeruginosa* and *Citrobacter freundii* were each isolated once. No patients were found to have polymicrobial infections. Among the 10 bacterial infections, seven were bloodstream and three were urinary. Only bloodstream infections (BSIs) were evaluated in subsequent analysis.

**Table 2.** Characteristics of 13 isolates from febrile individuals, including infection source and antibiotic resistance pattern

Patient	Isolate	Source	CHL	CLI	TET	SXT	AMP	AMC	CIP	GEN	NAL	CRO	CAZ	IPM	TZP
1	*S. pneumoniae*	Blood	R	S	R	X	S	S	X	X	X	S	X	X	X
2	*S. pneumoniae*	Blood	S	S	R	R	S	X	X	X	X	X	X	X	X
3	*P. falciparum*	Blood	N/A												
4	*P. falciparum*	Blood	N/A												
5	*E. coli*	Blood	S	X	X	R	R	R	R	R	R	S	S	S	X
5	*E. coli*	Blood	S	X	X	R	R	R	R	R	R	S	S	S	X
5	*C. freundii*	Urine	X	X	X	R	R	R	R	R	R	S	S	S	X
6	*P. aeruginosa*	Blood	X	X	X	X	X	X	X	X	X	X	S	S	S
7	*E. coli*	Blood/urine	R	X	X	R	R	R	R	X	R	R	R	S	S
7	*E. coli*	Blood/urine	R	X	X	R	R	R	R	X	R	R	R	S	S
7	*E. coli*	Urine	R	X	X	R	R	R	R	X	R	R	R	S	S
7	*E. coli*	Urine	R	X	X	R	R	R	R	X	R	R	R	S	S
8	*P. falciparum*	Blood	N/A												

Culture isolates were either susceptible (S), resistant (R) or not tested (X). AMP, ampicillin; AMC, amoxicillin/clavulanic acid; CAZ, ceftazidime; CRO, ceftriaxone; CHL, chloramphenicol; CIP, ciprofloxacin; CLI, clindamycin; GEN, gentamicin; IPM, imipenem; N/A, not applicable; NAL, nalidixic acid; TZP, piperacillin/tazobactam; TET, tetracycline; SXT, trimethoprim/sulfamethoxazole.

All six *E. coli* strains were resistant to ciprofloxacin, ampicillin, chloramphenicol, ampicillin/clavulanic acid and trimethoprim/sulfamethoxazole. The *S. pneumoniae* isolates were resistant to tetracyclines and susceptible to penicillin, erythromycin, ceftriaxone and clindamycin. *C. freundii* was resistant to quinolones but susceptible to third-generation cephalosporins. *P. aeruginosa* was susceptible to all antimicrobial agents tested.

### Risk factors and mortality

All 15 patients who experienced a febrile episode had a diagnosis of a haematological cancer; no patients with solid cancer experienced a febrile episode. There was no statistically significant relationship between HIV and likelihood of fever (6/26 HIV+ versus 9/24 HIV−; *P* = 0.36). There was no statistically significant relationship between HIV status and confirmed bacteraemia or infection (3/26 HIV+ versus 5/24 HIV−; *P* = 0.46). There was also no significant association between any of the presenting symptoms or neutropenia or severe neutropenia and the likelihood of detecting bacteraemia.

Out of the 23 febrile events, we only saw one death (case fatality rate = 4%). The one death was attributed to *Pseudomonas* bacteraemia that progressed to septic shock. Given there was only one death, further analyses of risk factors for death were not felt to be prudent. The patient who expired would not have met qSOFA criteria on admission, with blood pressure of 105/55 mmHg, respiratory rate of 22 breaths per minute, and no encephalopathy.

## Discussion

This study represents one of the first prospective investigations focusing on febrile events among cancer patients undergoing chemotherapy in SSA. Within our cohort at KCH, we documented a substantial incidence of febrile episodes, reaching 30% of enrolled patients among an admittedly high-risk patient population of haematological malignancies and/or persons with HIV and cancer. Though the sample size was small, we did confirm haematological malignancies as a risk factor for fever and a trend towards the same for HIV infection.

The rate of successful isolation of pathogens from febrile events was 13/23 (56%), with a bacteraemia rate of 10/23 (43%), both of which are high compared with similar studies. Comparable studies conducted in both paediatric and adult populations in Africa have reported bacteraemia rates from febrile episodes ranging from 14% to 22%.^16–19^ This may be due to the prospective nature of this study including both protocolized investigations and significant education efforts encouraging patients to regularly monitor their temperatures with a study-provided thermometer and return directly to KCH as soon as a fever of >38°C was detected.

Among the pathogens isolated in our cohort, Gram-negative bacteria (62%) emerged as the predominant pathogens, followed by *Plasmodium falciparum* (23%). This notable prevalence of Gram-negative bacteria as the primary causative agents of infection in cancer patients aligns with findings from Uganda (67%) and other low- and middle-income countries (LMICs).^23–26^ Conversely, in HICs, Gram-positive infections are more frequent in cancer populations, likely attributed to the frequent use of CVCs and prophylactic antibiotics targeting Gram-negative pathogens.^27^ Notably, two studies conducted on paediatric cancer patients in South Africa, where most patients had CVCs, yielded conflicting findings on this relationship between CVCs and infection with Gram-negative bacteria. One study found less CVC-related infection with more Gram-negative bacteria, while the other study found a higher risk of CVC-related infections.^17,19^ Critically, CVCs are not used in Malawi for treatment of cancer patients and therefore our finding of largely Gram-negative infections is in keeping with this trend despite frequent G-CSF and prophylactic antibiotic use.

Malaria was also a frequent cause of fever in patients undergoing chemotherapy in our population, occurring in three (6%) participants. SSA is a largely malaria-endemic region. In 2020 alone, Malawi reported over 4.4 million malaria cases.^28^ It is noteworthy that studies investigating NF are seldom conducted in LMIC or endemic malaria settings, and there exists a scarcity of research highlighting the risk of malaria during chemotherapy.^29^ Based on the findings of our study, it may be important to consider malaria as a potential causative agent in febrile episodes for patients undergoing chemotherapy in malaria-endemic regions. Fortunately, early detection of malaria allows for effective treatment and all patients with malaria in this study were treated successfully without complications.

Our study found a high burden of AMR among cancer patients in Malawi. AMR poses a significant challenge in SSA, and was responsible for an estimated 1.27 million deaths in SSA in 2019.^28^ Among *E. coli* isolates in our study, all were resistant to multiple antibiotics, with 4/6 (67%) being resistant to third-generation cephalosporins. Specifically, *E. coli* strains demonstrated complete resistance to aminopenicillins, fluoroquinolones and aminoglycosides. The high prevalence of resistance in *E. coli* among our cancer population mirrors findings in Uganda, where MDR and ESBL rates were described in 85% and 41% of isolates, respectively.^16^ Four strains of *E. coli* were susceptible to carbapenems and piperacillin/tazobactam, while the remaining two strains exhibited susceptibility to ceftriaxone as well. No carbapenem-resistant isolates were detected in this study but they are circulating in the community as recent AMR data from Malawi shows that the frequency of carbapenem-resistant *E. coli* in Malawi rose from 1% in 2017 to 41% in 2020.^30^ The management of MDR infections poses significant challenges due to limited therapeutic options, especially in SSA. Carbapenems are the preferred choice of treatment in both LMICs and HICs. However, increasing carbapenem-resistance rates is a cause for alarm, especially within high-risk populations and in settings where antimicrobial formularies may be restricted and decision-makers may consider tailored empirical therapy for a Malawi antibiogram, especially consideration of fourth-generation cephalosporins or piperacillin/tazobactam, given the possibility of *Pseudomonas* and frequent ceftriaxone resistance.

The sole death in our study was caused by *Pseudomonas* bacteraemia. While the AMR patterns of the *Pseudomonas* isolate did not show resistance to the drugs tested (ceftazidime, piperacillin/tazobactam and meropenem), the patient likely succumbed due to ineffective treatment with ceftriaxone. Malawian guidelines call for ceftriaxone and ciprofloxacin as the first-line therapy for sepsis, with escalation if there is no response.^28^ Unfortunately, this patient expired before antimicrobial susceptibility data were available to appropriately tailor antibiotics. Furthermore, given that there was only one death in this study, we were not able to adequately assess risk factors for death in this population, and limited vital sign data after hospital admission also limited our ability to investigate trends of deterioration. Continuous quality improvement programmes and antibiograms to tailor empirical antibiotic selection have been used successfully to decrease infectious morbidity and mortality among cancer patients in HICs and likely would also have similar value in SSA.^24^

Notably, haematological malignancies accounted for all instances of febrile and bacteraemic episodes in our study. These findings are consistent with research conducted in Uganda by Lubwama *et al.*, which demonstrated that 74% of bacteraemia cases were attributed to haematological malignancies, a pattern observed in HICs as well.^31,32^ Patients diagnosed with haematological malignancies tend to exhibit greater immunosuppression and receive more intensive cytotoxic chemotherapy compared with those with solid cancers, thereby resulting in higher rates of bacteraemia.^31,33^ Our study differed from that by Lubwama *et al.* in that the study in Uganda was conducted primarily in inpatients receiving intensive chemotherapy, whereas patients in our study were primarily outpatients. Despite this difference, findings are generally consistent across these settings.

Finally, we did not observe a correlation between HIV infection and NF, possibly due to limited sample size. In contrast, the rate of fever and confirmed infection have been reported to be higher from other LMICs.^34,35^ Neutropenia occurs frequently in patients with HIV infection, particularly in patients with haematological malignancies, which can lead to poor clinical outcomes.^36^

The strengths of our study include a prospective, standardized, detailed characterization of fever in cancer patients that allowed the identification of AMR and malaria. This was one of the first studies to characterize a prospective adult NF cohort where both malaria and HIV are endemic. Furthermore, providing patients with a thermometer for regular temperature monitoring and directing them to return to care upon fever occurrence likely increased the ascertainment of all fever episodes. Finally, including patients seeking routine care for a wide range of malignancies at a teaching hospital serving a large catchment area provides some generalizability despite the limited sample size. Limitations include the small sample size, the single-centre nature of the study, and minimal investigations for invasive fungal infections such as aspergillosis; future studies employing innovative molecular diagnostics would be insightful.^36^

### Conclusions

We describe a single-centre, prospective cohort of patients initiating chemotherapy with a high risk of NF at a national teaching hospital in Malawi. We observed a high incidence of fever and NF, with bacteraemia confirmed for a substantial proportion of these cases. The study also emphasizes the importance of malaria as a potential cause for febrile episodes during chemotherapy in SSA. Furthermore, the high prevalence of MDR *E. coli* in our study highlights the critical need for ongoing AMR surveillance and antibiotic stewardship to inform optimal prevention and treatment of NF in SSA. This is an example of the critical need for health system strengthening to improve cancer care; rapid and accurate data on AMR are critical for treating infections, both by preventing mortality from infectious complications and by allowing for chemotherapy intensification, which is so critical for effective cancer treatment. Larger studies are needed to identify pathogens most commonly associated with NF and inform effective prevention and treatment toward improving cancer treatment outcomes.

## Data Availability

Deidentified analytical datasets, study protocol and analysis code are stored in a password-protected repository and are available upon request to the corresponding author for reproducibility upon manuscript publication.
